# Radical Removal of Low-Differentiation Thymic Squamous Cell Carcinoma: A Rare Clinical Case

**DOI:** 10.7759/cureus.56370

**Published:** 2024-03-18

**Authors:** Boyko Yavorov, Vladimir Aleksiev, Petar-Preslav Petrov, Hristo Stoev, Zaprin Vazhev

**Affiliations:** 1 Cardiovascular Surgery, Medical University of Plovdiv, Plovdiv, BGR; 2 Human Anatomy, Histology and Embryology, Medical University of Plovdiv, Plovdiv, BGR

**Keywords:** vessel grafting, thoracic surgery, sternotomy, robotic surgery, thymic carcinoma

## Abstract

The presented case report demonstrates the successful operative treatment of a patient with thymic carcinoma located in the anterior mediastinum, infiltrating the vena cava, and affecting the upper lobe of the left lung. Our multidisciplinary approach, incorporating various operative techniques, proved effective in treating this type of pathology. The Clinic for Thoracic Surgery at UMHAT Kaspela, Plovdiv admitted a 72-year-old female patient due to complaints related to her cardiovascular and respiratory systems. The patient presented with symptoms such as chest pain, shortness of breath, cough with expectoration, and the presence of blood in her sputum. Additionally, the patient exhibited an increased temperature and experienced shortness of breath at rest. Extensive imaging and diagnostic studies were performed, including computed axial tomography of the chest with contrast material, echocardiography, functional breathing tests, and laboratory tests. The clinical board unanimously agreed that operative treatment was necessary, and the techniques used included robot-assisted surgery and median sternotomy. A low-differentiated carcinoma was identified during the surgical intervention and confirmed through patho-anatomical examination (frozen section) and permanent histological preparation. Immunohistochemical examination revealed that the immunophenotype of the tumor corresponds to thymic neuroplastic squamous cell carcinoma (poorly differentiated). The patient had a smooth postoperative period and was discharged in a satisfactory general condition.

## Introduction

Our team presents a clinical case of poorly differentiated thymic squamous cell carcinoma, which holds fundamental importance for clinical practice [1]. This significance is due to the application of a multidisciplinary approach during operative interventions, incorporating a combination of various operative techniques [2]. Thymic carcinoma is a tumor originating from the thymus gland. These carcinomas are oncological malignancies with a potential for metastasis to neighboring organs and the possibility of distant metastases [3]. The symptoms of thymic carcinoma vary depending on the size of the tumor and may affect the cardiovascular and respiratory systems.

## Case presentation

**Figure 1:**
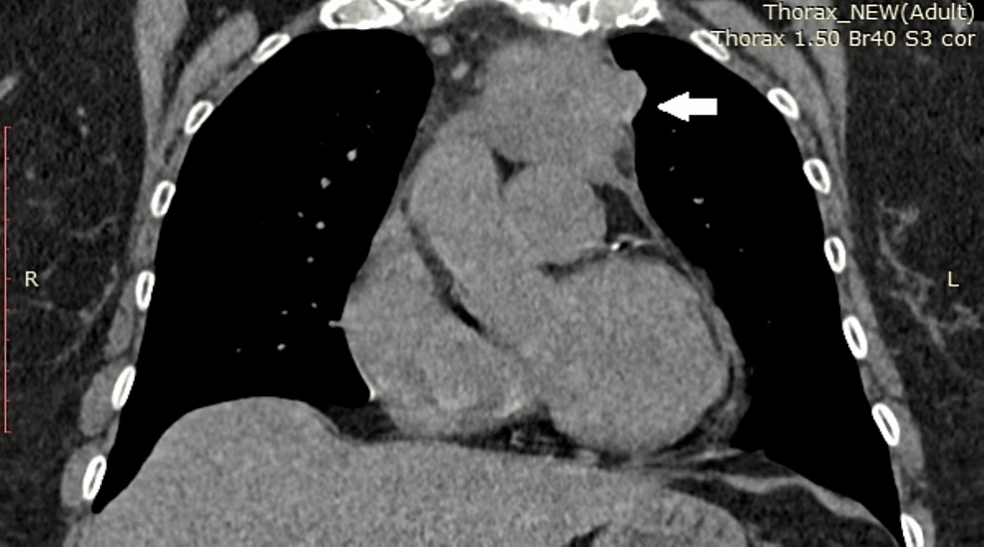
Preoperative computed axial tomography. The formation is indicated by an arrow. A borderline lymph node up to 10 mm in size is shown near the left truncus pulmonalis, with no pericardial effusion.

**Figure 2:**
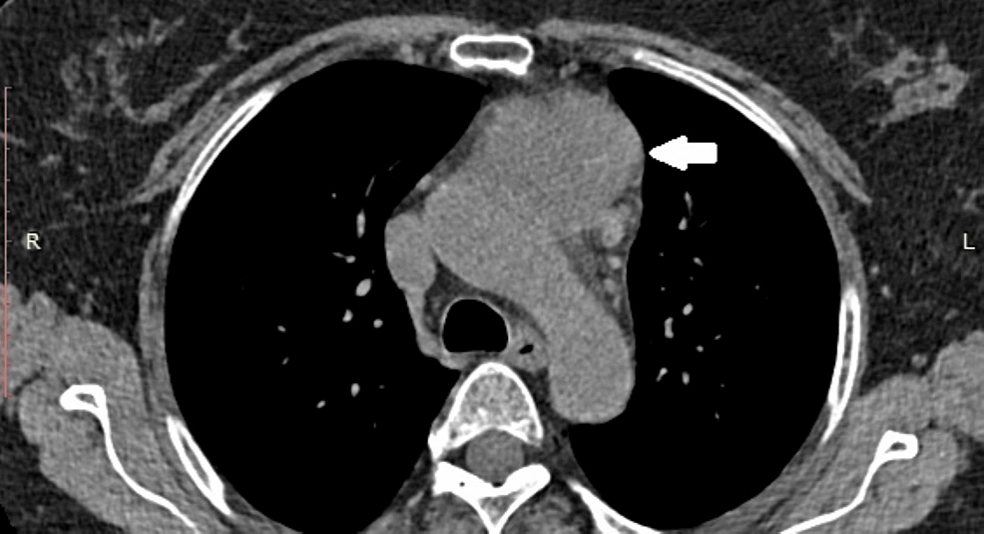
Preoperative computed axial tomography: The formation, indicated by an arrow in the superior mediastinum in front of the aortic arch on the left, is a lobulated soft tissue formation. It has densities of about 30/35 Hounsfield units and transverse dimensions of approximately 4.35 cm by 5.00 mm. This formation is intimately adjacent to the arch of the aorta and extends to the truncus pulmonalis.

The patient is a 72-year-old female who was admitted to the Clinic for Thoracic Surgery at UMHAT Kaspela, Plovdiv, due to complaints related to the cardiovascular and respiratory systems. The anamnestic symptomatology includes heaviness and pain in the chest, tightness behind the sternum, shortness of breath with minimal physical effort, cough with copious expectoration, the presence of clear blood in the sputum, an increase in temperature up to 38 degrees Celsius, as well as manifestations of shortness of breath at rest. The objective condition of the patient: a woman appearing her stated age, in satisfactory general condition, contactable and adequate. Subfebrile temperature up to 37.5 degrees Celsius. Peripheral lymph nodes were not enlarged upon palpation. Examination revealed a normal status of the cardiovascular system and chest. Succusio renalis was negative bilaterally. The limbs showed no pretibial edema and mobility was preserved. Our team conducted image-diagnostic studies. Computed axial tomography of the chest with contrast material revealed a lesion in the superior mediastinum in front of the aortic arch to the left - a lobulated soft tissue formation with densities of about 30/35 Hounsfield units and transverse dimensions of about 4.35 cm by 5.00 mm, intimately adjacent to the arch of the aorta and extending to the truncus pulmonalis. Additionally, a borderline lymph node up to 10 mm in size near the left truncus pulmonalis, without pericardial effusion, was noted (Figures 1-2).

**Figure 3:**
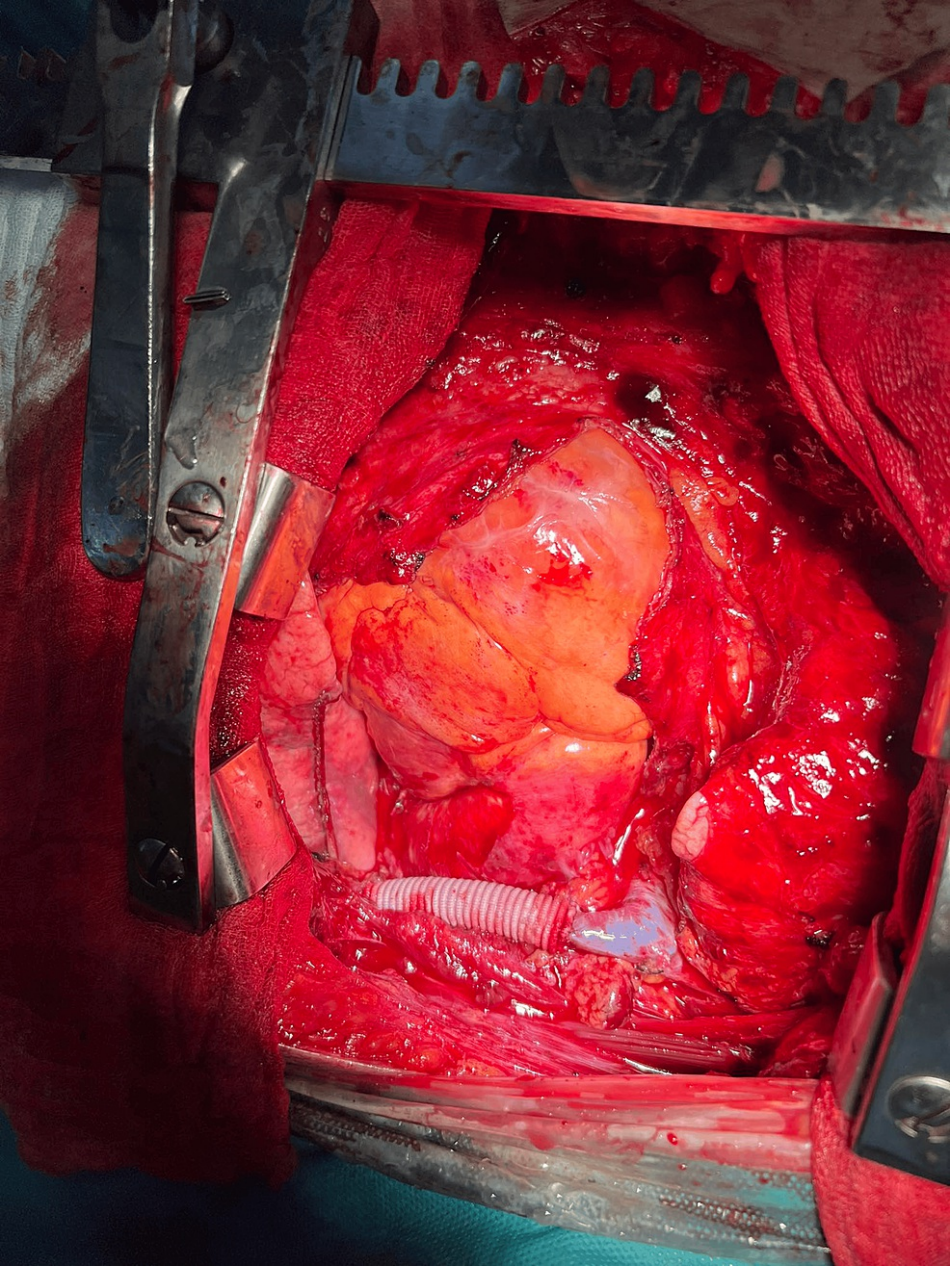
Intraoperative finding: The prosthetic brachiocephalic vein and the resected pericardium can be seen.

**Figure 4:**
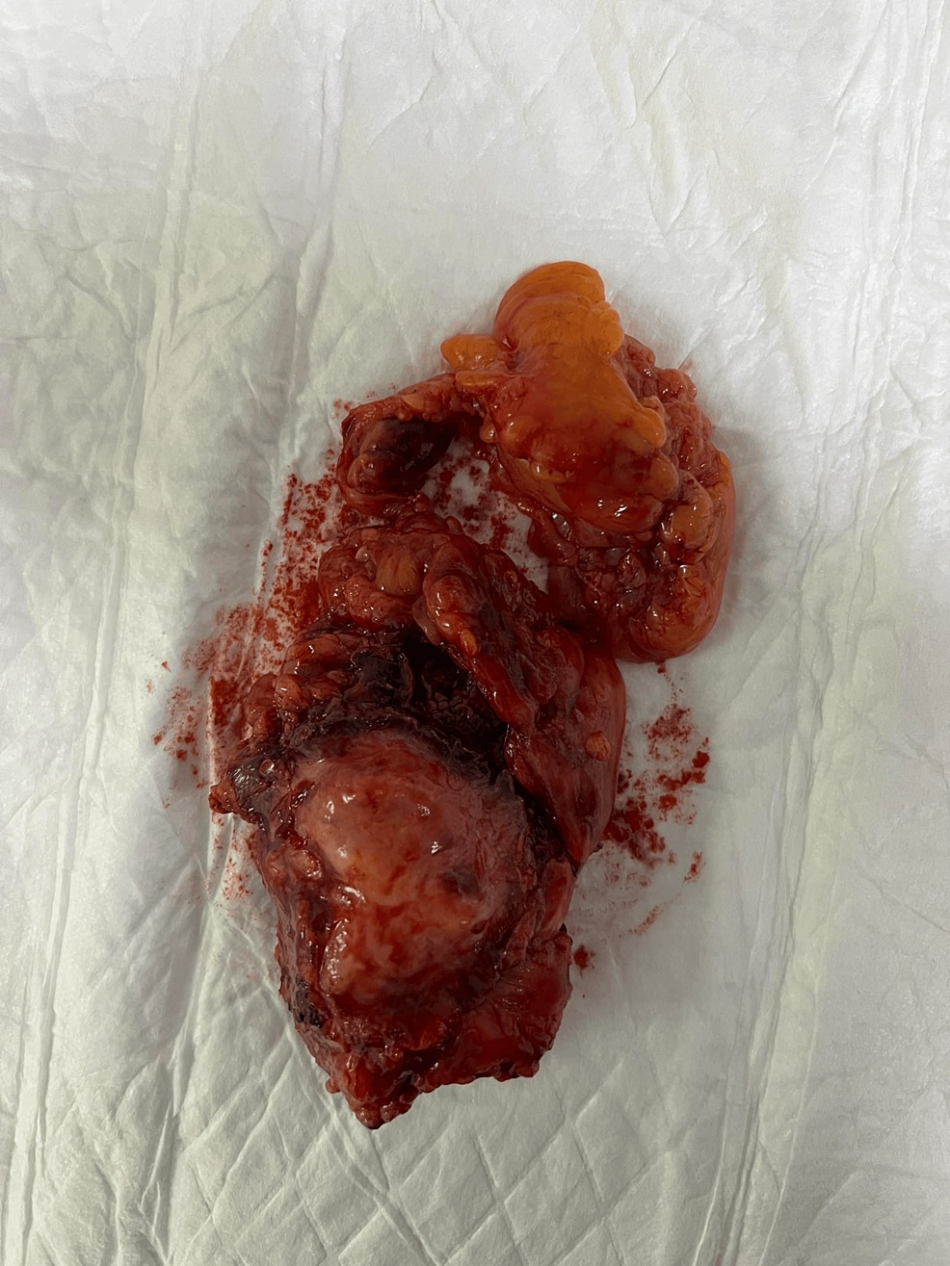
Intraoperative finding: The specimen is presented. The entire thymus, along with the tumor, was removed.

After extensive clinical discussion, we decided on surgical treatment for the presented pathology. Under general anesthesia and following thorough cleaning of the operative field, we initiated the procedure with three incisions made at typical anatomical sites in the thoracic region, through which three working ports were inserted. The three robotic arms of the DaVinci system were then docked. During the exploration, we discovered a tumor formation infiltrating the mediastinal pleura, the upper lobe of the left lung, and the left brachiocephalic vein (innominate). A careful dissection using the DaVinci system was performed to partially release the tumor and to dissect the mediastinal lymph nodes. Material was taken for a histopathological examination - a frozen section, which confirmed a poorly differentiated carcinoma. Given the involvement of the mentioned structures and the impossibility of their radical removal with the DaVinci system, we proceeded with a median sternotomy. We performed an atypical resection of the upper lobe of the left lung using an automatic stapler. The pericardium was resected, and the tumor formation was sharply separated from the adventitia of the aorta and the pulmonary trunk. The phrenic nerve, infiltrated by the tumor, was also resected. Due to tumor infiltration, a 5 cm section of the innominate vein was resected, and a vascular prosthesis was implanted following heparinization of the patient, with the interposition of a 10 mm vascular prosthesis (Figures 3-4).

**Figure 5:**
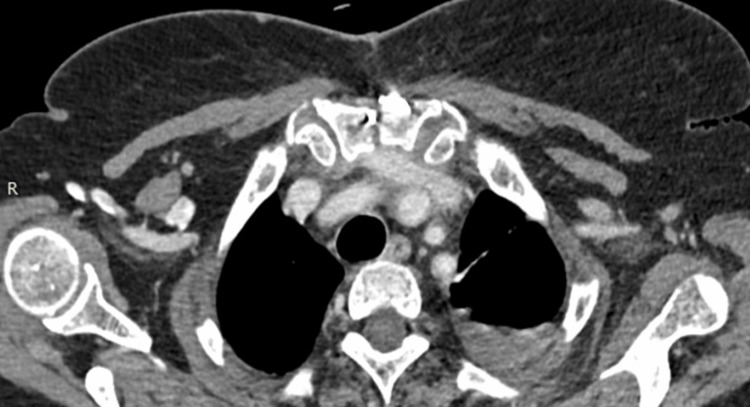
Postoperative computed axial tomography: The control CT scan with contrast material shows that the prosthetic brachiocephalic vein is patent and both lungs are fully dilated.

Postoperatively, we transferred the patient to the Department of Anesthesiology and Intensive Care with mechanical ventilation, hemodynamically stable and without the need for catecholamine support. Extubation was performed on the first postoperative day. The operative wound was clean, with no evidence of active bleeding from the drains. On the second postoperative day, after transferring the patient to the Clinic of Thoracic Surgery and performing an air leak test, the left pleural drain was removed, followed by the removal of the two retrosternal drains on the third postoperative day. Before discharging the patient, we conducted a control computed axial tomography with contrast material and found that the prosthesis was in good condition and patent. During the operation, we administered Heparin for grafting. Subsequently, still intraoperatively, we administered Protamine sulfate. Postoperatively, the patient was prescribed acenocoumarol tablets for six months, followed by an antiplatelet drug (Figure 5).

**Figure 6:**
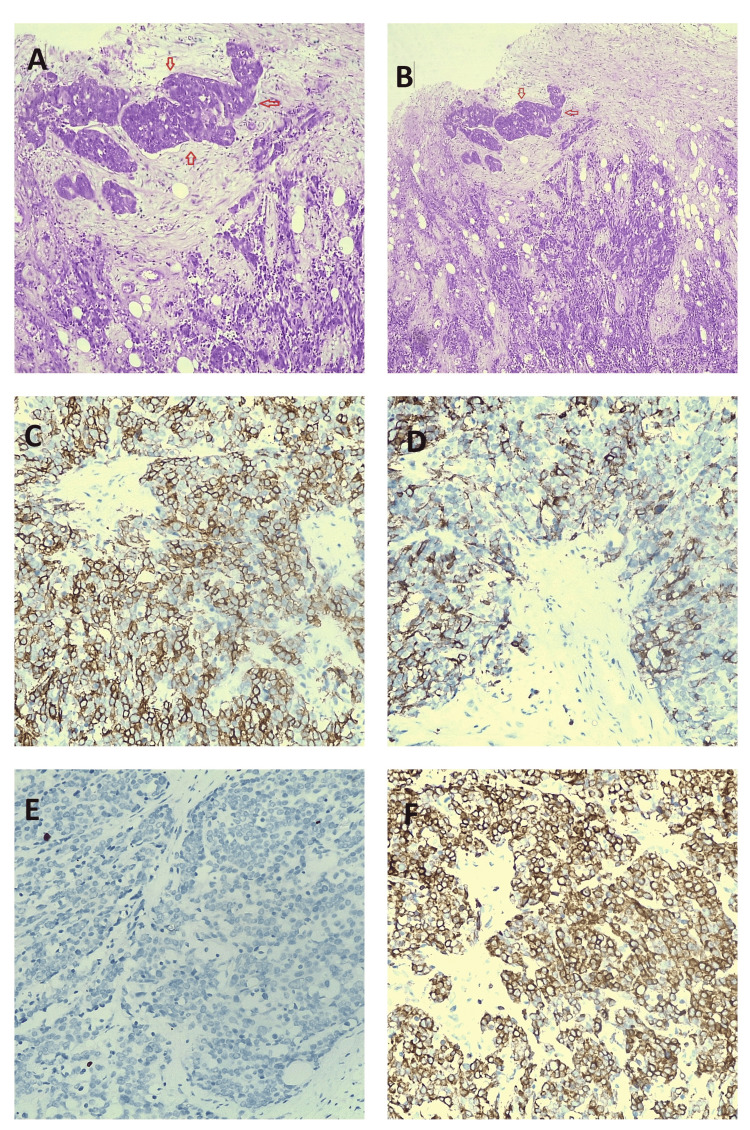
Thymic squamous cell carcinoma: (A) Irregular nests of tumor cells are observed growing in a desmoplastic stroma. (B) The tumor cells exhibit increased cytologic atypia, with slight nuclear pleomorphism, vesicular nuclear chromatin, and conspicuous nucleoli. (C) The tumor cells express GLUT1 diffusely (90% of tumor cells show membranous expression), CD5 (100% of tumor cell expression), and CD117 (90% of tumor cell expression).

Histological examination of the permanent preparation confirmed the findings of the frozen section. Immunohistochemical analysis of the specimen established that the immunophenotype of the tumor corresponded to a poorly differentiated thymic squamous cell carcinoma (Figure 6).

We performed the standard mediastinal lymph node dissection and sent all the lymph nodes for histopathological examination; they came back negative. After discharging the patient, she was considered for chemotherapy and immunotherapy. The patient was discharged in a satisfactory general condition.

## Discussion

Thymic carcinomas are characterized by the potential for metastasis to both nearby and distant organs and structures. The main symptomatic manifestations of the disease vary and depend on both the degree of advancement of the process and the size of the tumor, as well as its relationship to neighboring structures and organs [4]. The primary complaints arise from the cardiovascular and respiratory systems. This type of pathology occurs relatively infrequently in the population and affects men and women at the same percentage ratio. After a clinical consultation, our team opted for surgical treatment using the Da Vinci robotic system, which offers the advantages of reduced trauma, lower intraoperative and postoperative bleeding, and a shorter recovery period for the patient [5]. During the surgical intervention, due to the established infiltration of the anonymous vein and the upper lobe of the left lung, a median sternotomy was performed to better expose the structures, control the large vessels, and achieve radical removal of the tumor [6]. Globally, and according to the literature, video-assisted and robot-assisted surgery is the main operative method for types A, AB, B1, B2, and B3 thymomas [7]. The video-assisted operative technique may include unilateral video-assisted thoracoscopic (VATS), transcervical VATS, and subxiphoid VATS. However, these methods are not suitable for thymic carcinomas and some large B3 thymomas with involvement of adjacent organs and structures, due to the impossibility of their radical removal. Median sternotomy, which remains the method of choice for thymic carcinomas, can provide adequate exposure of the mediastinal structures, sufficient space for surgical manipulations, and control of the great vessels [8]. For thymic carcinomas infiltrating the heart, an operative technique employing a cardiopulmonary bypass machine (extracorporeal blood circulation) can be applied [9], offering an advantage in the radical removal of the tumor and improving patient survival.

## Conclusions

In established thymic carcinoma, a positive outcome relies on an accurate diagnosis and effective communication among members of the multidisciplinary team, aiming for timely operative treatment. Surgical treatment is the primary method of choice for this type of pathology and is particularly effective in the early stages of thymomas, which correlates with a higher survival rate. The choice of surgical technique depends on the disease's progression, the tumor's size and its relationship to neighboring tissues and organs, and, most importantly, the team's expertise. Due to the risk of recurrence, postoperative follow-up of patients is recommended.
